# Testing the effects of narrative and play on physical activity among breast cancer survivors using mobile apps: study protocol for a randomized controlled trial

**DOI:** 10.1186/s12885-016-2244-y

**Published:** 2016-03-09

**Authors:** Elizabeth J. Lyons, Tom Baranowski, Karen M. Basen-Engquist, Zakkoyya H. Lewis, Maria C. Swartz, Kristofer Jennings, Elena Volpi

**Affiliations:** Department of Nutrition and Metabolism, The University of Texas Medical Branch, 301 University Blvd, Galveston, TX 77555-1124 USA; USDA/ARS Children’s Nutrition Research Center, Baylor College of Medicine, 1100 Bates St, Houston, TX 77030 USA; Department of Behavioral Science and Center for Energy Balance in Cancer Prevention and Survivorship, M.D. Anderson Cancer Center, 1155 Pressler St, Houston, TX 77030 USA; Division of Rehabilitation Sciences, The University of Texas Medical Branch, 301 University Blvd, Galveston, TX 77555 USA; Department of Preventive Medicine and Community Health, The University of Texas Medical Branch, 301 University Blvd, Galveston, TX 77555 USA; Department of Geriatrics and Claude D. Pepper Older Americans Independence Center, The University of Texas Medical Branch, 301 University Blvd, Galveston, TX 77555 USA

**Keywords:** Physical activity, Breast cancer, Cancer survivorship, Video games, Narrative, mhealth, Intervention, Mobile app

## Abstract

**Background:**

Physical activity reduces risk for numerous negative health outcomes, but postmenopausal breast cancer survivors do not reach recommended levels. Many interventions encourage self-monitoring of steps, which can increase physical activity in the short term. However, these interventions appear insufficient to increase motivation for sustained change. There is a need for innovative strategies to increase physical activity motivation in this population. Narratives are uniquely persuasive, and video games show promise for increasing motivation. This study will determine the effectiveness of an intervention that combines narrative and gaming to encourage sustained physical activity.

**Methods/Design:**

SMARTGOAL (Self-Monitoring Activity: a Randomized Trial of Game-Oriented AppLications) is a randomized controlled intervention trial. The intervention period is six months, followed by a six month maintenance period. Participants (overweight, sedentary postmenopausal breast cancer survivors aged 45–75) will be randomized to a self-monitoring group or an enhanced narrative game group. The self-monitoring group will be encouraged to use a mobile application for self-monitoring and feedback and will receive 15 counseling phone calls emphasizing self-regulation. The narrative game group will be encouraged to use a mobile application that includes self-monitoring and feedback as well as a narrative-based active video game. The 15 calls for this group will emphasize concepts related to the game storyline. Counseling calls in both groups will occur weekly in months 1 – 3 and monthly in months 4 – 6. No counseling calls will occur after month 6, but both groups will be encouraged to continue using their apps. The primary outcome of the study is minutes of moderate to vigorous physical activity at six months. Other objectively measured outcomes include fitness and physical function. Self-reported outcomes include quality of life, depression, and motivation.

**Discussion:**

This protocol will result in implementation and evaluation of two technology-based physical activity interventions among breast cancer survivors. Both interventions hold promise for broad dissemination. Understanding the potential benefit of adding narrative and game elements to interventions will provide critical information to interventionists, researchers, clinicians, and policymakers. This study is uniquely suited to investigate not just whether but how and why game elements may improve breast cancer survivors’ health.

**Trial registration:**

clinicaltrials.gov NCT02341235 (January 9, 2015)

**Electronic supplementary material:**

The online version of this article (doi:10.1186/s12885-016-2244-y) contains supplementary material, which is available to authorized users.

## Background

Breast cancer is the most prevalent cancer among women worldwide [1]. Breast cancer survivors represent a large population that faces unique challenges to health and well-being from their cancer, its treatment, and from co-morbidities. Physical activity is an increasingly recognized method to address many of these challenges. Physical activity improves mood [2, 3], physical functioning [4], and pain [4], leading to improvements in quality of life and fatigue [5, 6]. Both physical activity duration and intensity appear to have a dose–response relationship with improved health [7], but improvements can result from as little as 15 minutes per day of moderate-intensity activity [7–10]. While 150 minutes or more of moderate intensity physical activity per week has been associated with decreased breast cancer recurrence and mortality as well as improved quality of life [11–14], only 37 % of breast cancer survivors reported meeting this recommendation [15]. Objective measures of activity revealed that breast cancer survivors accumulated approximately four minutes per day of moderate to vigorous intensity activity, compared to eleven minutes in non-breast cancer controls [16]. This difference was sustained years after diagnosis and treatment [17, 18].

Cancer mortality was higher among African-American than among White women [19], and disease-free survival, quality of life, and physical function were lower in African-American and Hispanic women [20, 21]. Though physical activity could produce beneficial effects for survival, comorbidities, and quality of life, minority breast cancer survivors were less active than White survivors [20, 22]. Therefore, interventions are needed that appeal to a broad range of women, in particular African American and Hispanic survivors.

Interventions for increasing physical activity among cancer survivors typically include pedometers to self-monitor walking along with behavioral counseling to deliver behavior change techniques [23, 24]. These interventions have been effective in the short-term, but adherence decreased quickly [25]. For example, by week 9 of a 12-week intervention, only 53 % of participants reached their activity goals [26]. Assessment of behavior maintenance in the absence of further investigator contact is rare in physical activity studies, particularly among breast cancer survivors [27]. Secondary investigations of intervention trials and review papers have suggested that targeting autonomous motivation may be one of the most promising methods for maintaining activity among survivors [28, 29]. Thus, current approaches appear to be inadequate for producing sustained increases in physical activity, and little is known about how behaviors change once investigator contact ceases. Increasing autonomous motivation to be active is a promising strategy for improving intervention effectiveness and maintenance thereof. Innovative techniques are needed to target motivation and ensure intervention components motivate women of all races and ethnicities.

### Theoretical framework

This intervention is predicated on constructs from Self-Determination (SDT) and Narrative Transportation Theories (NTT). SDT proposes a continuum between autonomous and controlled behavioral regulations or motivations [30, 31], with autonomous motivation being more strongly associated with physical activity over time [32, 33]. Autonomous motivation for physical activity includes both completely intrinsic motivation (motivation due to inherent interest and enjoyment) as well as integrated and identified regulation (relatively autonomous forms of extrinsic motivation that involve internalizing external motivations and valuing the outcomes of activity, respectively). Autonomous motivation is increased by the fulfillment of psychological needs for competence, autonomy, and relatedness [30]. Several large-scale autonomy-promoting interventions have demonstrated long-term maintenance of physical activity [34] and weight loss [35, 36].

Narrative transportation, i.e. absorption into a story, can influence both autonomous motivation and beliefs about health behaviors, such as cancer screening [37]. Transportation has been associated with media enjoyment and attentional focus [38], which were associated with positive affect and motivation [39]. Breast cancer survivors who watched a video of other survivors telling their stories, compared to those who watched an informational video, reported feeling more attentive and less upset [40]. Narratives reduced counter-arguing as compared to standard information provision [41], which led to greater attitude change. Narrative transportation may even affect individual perceptions of identity and belonging [42].

**Fig. 1:**
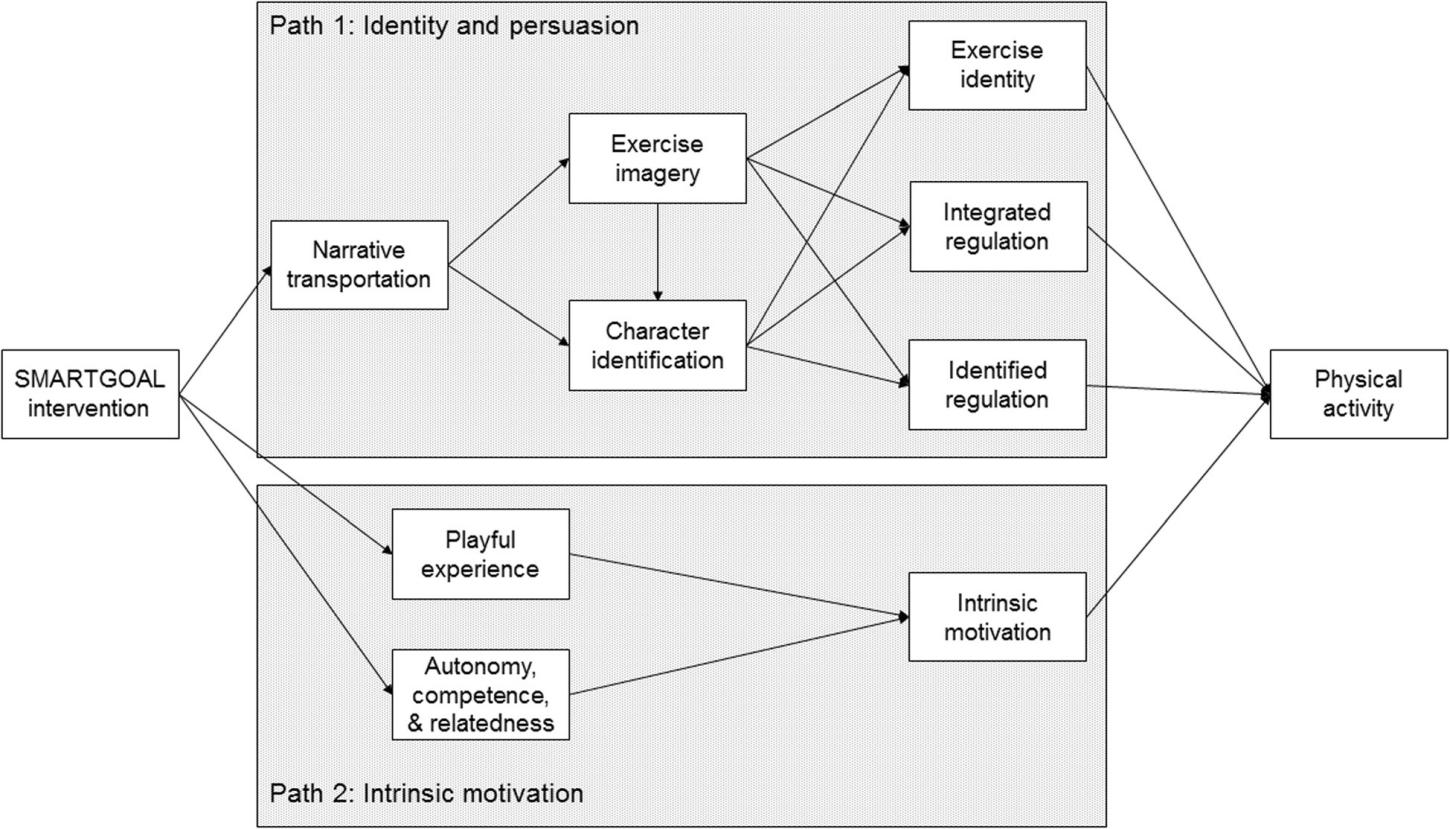
SMARTGOAL conceptual model with relationships of interest

This study will test a conceptual model that consists of two complementary pathways: one involving narrative, identity, and persuasion, and another involving playfulness and intrinsic motivation. An active video game that includes both narrative and fun elements should impact each of the pathways, which would ultimately lead to adoption of the desired behavior, physical activity. Figure 1 displays the major relationships of interest.

#### Narrative transportation pathway

The identity and persuasion pathway involves narrative transportation influencing participants’ beliefs about themselves. We hypothesize that narrative transportation in the game will increase exercise imagery and character identification, leading to changes in identity and values.

Exercise imagery is typically operationalized by asking individuals to imagine themselves exercising and to feel certain emotions related to exercise. Live-action role playing in a game context by its very nature should produce a substantial amount of imagery, as players are encouraged to actively imagine participating in the game world and events. Imagery scripts typically describe a workout, thoughts and feelings that might occur while exercising [43], and achieving goals via exercising [44]. Imagery (as a general concept) is also one of the theoretical components of narrative transportation [45]. Story imagery that involves exercise may function similarly to explicit exercise imagery interventions. Enjoyment and energy-related imagery have been linked to increased positive affect related to exercise [46]. Imagery related to technique and enjoyment were also related to autonomous motivation [47].

Character identification, as defined here, indicates a temporary shift in self-perception during which game players perceive their attributes as similar to those of the character they play [48]. In other words, character identification offers an opportunity to “try on” new characteristics and values [49], potentially reducing discrepancy between the current self and the imagined ideal self [50]. Video gaming resulted in strong identification effects [48, 49], perhaps due to the explicit role-playing and “experience-taking” [51] that occurs during video game play, compared to reading or listening to a story. Character identification has been associated with both narrative transportation and behavior change [52, 53].

We hypothesize that exercise imagery and character identification will also affect two forms of autonomous motivation: identified regulation and integrated regulation. For example, an individual would exercise because they value its outcomes (identified regulation) and because they value exercise itself (integrated regulation). Also, by experiencing an identity that values fitness and strength while pretending to be a game character, players may come to value fitness and strength.

The identity and persuasion pathway may be particularly important for minority race/ethnicity women. Culturally appropriate storytelling persuaded ethnic minority women to be screened for cancer [41]. Narratives that included characters similar to the reader/listener enabled character identification and thus persuasion [54]. Video games are excellent vehicles for such storytelling because they can provide racially ambiguous and/or customizable characters that are similar to a broad range of players.

#### Intrinsic motivation pathway

The intrinsic motivation pathway hypothesizes a causal relationship such that playful experience and perceptions of autonomy, competence, and relatedness influence intrinsic motivation. A playful experience is one that is perceived as emphasizing freedom, focused attention, and safety from real-world consequences [55]. All play is in essence practice because it is viewed as having fewer consequences than real-life behaviors [56]. Games encourage experimentation with difficult behaviors by making failures expected on a challenging trajectory towards mastery, framing failure as a state rather than a trait – unstable, specific, and within one’s capacity to change [57].

Video games are also effective tools for influencing the basic psychological needs of autonomy, competence, and relatedness, in turn increasing intrinsic motivation [58]. Greater intrinsic motivation, or the similar concept of enjoyment, has been linked to greater physical activity over time in studies of game-based cycling [59] and to greater energy expenditure in laboratory studies of cycling and console active games [60, 61]. Even in a game with a pre-determined narrative, players can feel autonomy by making choices and via representational agency (e.g., feeling powerful because they are role-playing as someone powerful) [57]. Games support competence by providing guided practice opportunities to work towards mastery. Non-player characters in games, much like supporting characters in books, can provide social influence and feelings of belonging [42].

### Aims and objectives

This project will test the effectiveness of an intervention that uses narrative and play elements coupled with a standard self-monitoring mobile intervention on physical activity. We hypothesize that the narrative game intervention will produce greater physical activity at the conclusion of the intervention period (6 months) as well as at 1 year (6 months of maintenance) than self-monitoring alone. We will also explore the effects of the intervention on other physiological outcomes (fitness, function, weight) and self-reported outcomes (quality of life, depression, fatigue, anxiety, sleep), hypothesizing that the narrative game intervention will also produce greater positive effects on these and intermediate motivation-related variables.

## Methods/Design

### Preliminary focus group and pilot intervention data

#### Focus groups and interviews

Prior to carrying out a pilot study, it was necessary to determine basic feasibility of the technologies to be used. Focus groups and interviews with 20 female breast cancer survivors 55 – 79 years old were conducted to investigate what kinds of mobile devices and games were considered usable and acceptable in this population. A convenience sample was recruited using newspaper and email list solicitations, and meetings occurred in common meeting locations for breast cancer survivors (e.g., space used for support group meetings). Transcripts were analyzed using thematic analysis principles [62].

When asked about video games, the women reported a lack of general knowledge. However, many of those same women also discussed playing and enjoying games such as *Angry Birds*, *Farmville*, and *Lumosity*, which they did not consider video games. Seven of the women mentioned experience playing active video games using a Nintendo Wii console, and nearly all stated that they were interested in trying active games if someone explained how to play.

The women were very skeptical of smartphones, but enthusiastic about tablets. They commonly mentioned simplicity as the most important aspect of any technology, and they perceived tablets as simpler than smartphones (perhaps due to comparisons – tablets are simple in comparison to computers, whereas smartphones are complicated in comparison to regular phones).

When asked specifically about their preferences for an active game, the women mentioned greatly enjoying music. However, they worried that the dance games we showed them (*Dance Central* and *Just Dance*) were too difficult or too complicated. There was substantial enthusiasm for games that involved pretending to be in interesting places. When questioned specifically about *Zombies, Run!*, the participants expressed skepticism initially about a zombie-themed game, but enjoyed the idea of a suspenseful story-based game once the specific concept was explained. A “Choose Your Own Adventure” style interactive narrative was also deemed interesting. They liked the idea of scavenger hunt games, but thought they would get bored with a game that only consisted of collecting resources.

#### Pre-experimental study of narrative-based mobile games

A 12-week pilot study was conducted to test basic feasibility and acceptability of a narrative-based mobile walking game. Ten adult women (52 ± 13 years old, 31 ± 4 BMI, sedentary and overweight) were recruited from the community using newspaper and online mailing list solicitations. Nine women completed the study, and one dropped out because she became pregnant. Participants were provided with mobile phones with the game *Zombies, Run!* (Six to Start, London, UK) pre-installed. They were also provided $55 for downloading music to use while playing the game. (The game will be described in detail in the intervention section below.) At the initial session, participants set goals for weekly walking. Weekly phone calls reviewed goals and briefly addressed other behavior change techniques based in self-regulation, such as self-monitoring, feedback, and problem-solving.

The major outcomes of this trial involved acceptability and feasibility difficulties due to loaning phones (iPhone 4, Apple, Inc., Cupertino, CA) to participants. Acceptability was measured by self-report questions previously used in similar studies [63, 64], scores on validated psychological measures, as well as open-ended feedback. Feasibility was operationalized as ability to use various aspects of the technology as intended.

All 10 participants completed questionnaires related to their feelings on the first *Zombies, Run!* mission. Narrative transportation ratings were relatively high (M = 47.6, SD = 9.77 using a 12-item version of the Narrative Transportation Scale with an 84 maximum possible score; items from this scale are mentioned in the measurement section below), even higher than in our past studies of women playing active video games, sedentary video games, and watching TV (means of 35, 37, and 44, respectively) [65]. Intrinsic motivation was rated a mean of 5.1 (SD = 1.2) out of 7, similar to intrinsic motivation ratings of active and sedentary video games and watching TV in the above-mentioned study (5.2 – 5.4) [65].

Acceptability measures adapted from previous similar interventions [63, 64] were recorded on 5-point Likert scales from 1 (strongly disagree) to 5 (strongly agree). None of the participants reported problems with the storyline or being turned off by the scary nature of the game. All participants reported that zombie chases encouraged them to go faster. Other acceptability ratings were lower than expected, although these ratings may be due to frustrations with the phones rather than the content of the mobile application (app). For example, of the nine women who completed the study, only four agreed or strongly agreed that they felt confident using the phone we provided. Six of nine women agreed or strongly agreed that the app was convenient to use and user-friendly.

Open-ended feedback from participants indicated that several of them disliked having to deal with two different phones, since they had phones of their own. Several participants stated that they preferred the option to use their own devices rather than be forced to use the loaner phone we provided. The game itself appeared to be acceptable, but the mobile device used for its delivery should be changed.

It was determined that protocol feasibility would also require refinements. Participants were harassed by collection agency and scam phone calls despite our best efforts to block numbers (91 calls across 10 phones), indicating that a device without phone functionality would likely be more usable. Connection of the *Zombies, Run!* game to the *Runkeeper* (Fitnesskeeper, Inc., Boston, MA) system for surveillance purposes was found to be feasible. Each participant’s *Zombies, Run!* account was connected to a matching *Runkeeper* account, which allowed investigators to view logs of the date and length of time of participants’ walks via *Runkeeper*’s online portal. Five participants used only the accelerometer, and four used both the accelerometer and GPS. It appears that an iPod Touch or smartwatch would be adequate to play the game and avoid some of the problems associated with the smartphone device.

We concluded that the game was likely feasible and appropriate if implemented using several protocol refinements: 1) allowing participants to use their own phones if they wish, 2) providing mobile devices that were not phones, such as an iPod Touch, and 3) ensuring that we provide sufficiently clear and simple technical instruction on use of the mobile devices and game.

### Participants and setting

SMARTGOAL (Self-Monitoring Activity: Randomized Trial of Game-Oriented AppLications) is a randomized controlled trial that will compare two different technology-based interventions. Participants will be randomized to either an intervention that enhances standard self-regulatory content with narrative and game elements, or to a standard intervention only providing self-regulatory content. We will recruit 120 breast cancer survivors to participate in the year-long study. Only orientation, initial counseling, and assessment contacts will take place in-person. Exercise will be self-paced and self-directed walking, with motivational assistance from one of two mobile applications (depending upon intervention assignment). All other investigator contact will occur via phone or the app assigned to that intervention group.

### Recruitment, screening, and randomization

We will recruit participants in three cohorts of 40 participants each. We anticipate recruiting a convenience sample of participants primarily via mailings based on registries of local older adult volunteers, individuals who have consented to be included in participant registries for aging studies, and lists of breast cancer patients who receive care at affiliated clinics (with consent and a signature from their physicians). We will also use direct solicitation at breast cancer-related events such as 5 k runs, survivorship conventions, support group meetings, and other social events. As needed, standard strategies such as newspaper advertisements, online mailing list emails, and flyers will be used.

Based on the racial and ethnic makeup of local counties [66] and recruitment into previous studies, we expect approximately 30 % or more of the sample to be of African American and/or Hispanic race/ethnicity. However, should the third cohort begin with a lower than expected proportion of underrepresented minority participants, we will preferentially recruit individuals based on a quota of 30 %.

Eligibility criteria will include self-reports of female gender, postmenopausal status (cessation of menses for at least 12 months), aged 45–75, current inactivity (<90 minutes moderate-vigorous activity per week), 25 < BMI < 40, no hospitalization for psychiatric problems in the past year, breast cancer diagnosis ≤ 10 years prior to recruitment, no chemotherapy, surgery or radiation treatment in the past six months, no evidence of disease recurrence, ability to walk for physical activity, and not currently using the application to be provided. The PAR-Q+ will be used to screen for potential contraindications to exercise [67]. Endorsement of any of its items will require physician clearance to participate in the study.

Participants will be randomized using sequentially numbered opaque sealed envelopes following standard procedures. Envelopes containing group assignments (obscured by aluminum foil and backed by carbon paper) will be shuffled and numbered sequentially then assigned to participants after each baseline assessment. The participant’s ID number, the date, and signature of the opener will be written on the envelope to provide an audit trail.

Blinding of participants to their group is not possible, but both will be receiving a technology-based intervention. Members of the assessment and evaluation team will be blinded to group assignment; interventionists will be conducting orientations, providing technical support, and conducting telephone counseling and thus cannot be blinded.

This protocol has been approved by the University of Texas Medical Branch Institutional Review Board and registered at clinicaltrials.gov (NCT02341235).

### Protocol

**Fig. 2:**
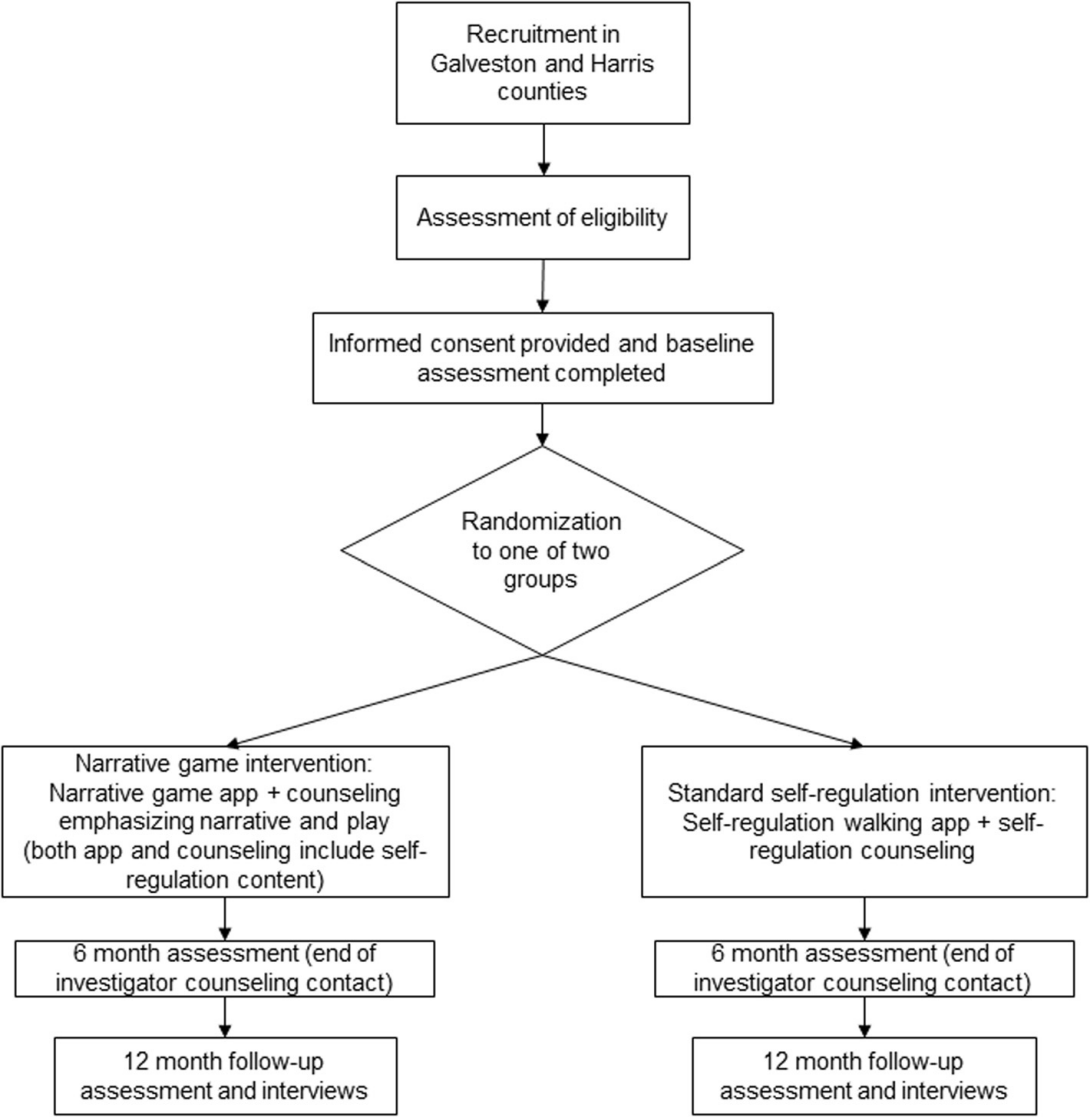
Study flow

The trial will be conducted over the course of three years, in three yearly cohorts. Participants in the trial will each attend four visits: a baseline assessment/orientation, an initial individual counseling/goal-setting meeting, a 6 month assessment, and a 12 month assessment. Visits will occur in University-owned clinical buildings in Galveston or Harris counties. Prior to the baseline assessment, potential participants will be provided with informed consent information in a private room and allowed time to read and ask questions as necessary. Only after signing an informed consent document approved by the Institutional Review Board will participants move on to assessment activities. Regular intervention contacts by phone (weekly for months 1 – 3, monthly for months 4 – 6) will occur for the first 6 months. The period between 6 and 12 months will be monitored as a maintenance period, to determine whether behaviors are maintained in the absence of further counseling. The schedule of enrollment, intervention, and assessment is shown in the format recommended by the SPIRIT (Standard Protocol Items: Recommendations for Interventional Trials) guidelines [68], which mirrors applicable items from the CONSORT 2010 guidelines [69], in Table 1. The flow of the study is shown in Fig. 2 Additional file 1 includes a completed CONSORT checklist. Any important changes to these methods will be discussed in final reports of this trial, with date and rationale for the changes.

**Table 1:**
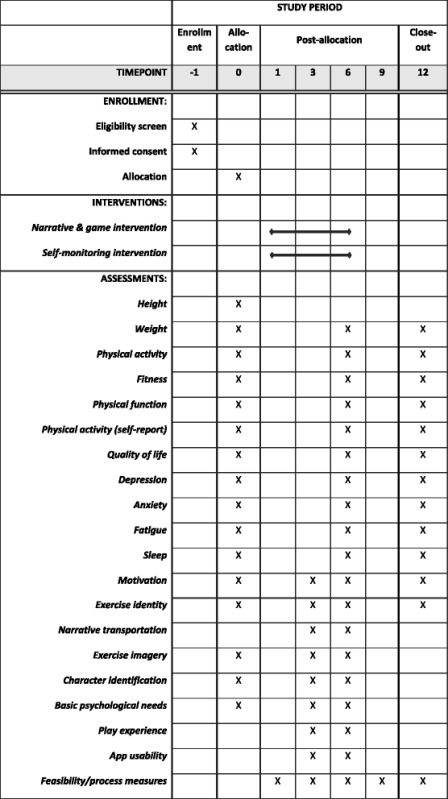
Schedule of enrollment, interventions, and assessments

All participants will receive $25 gift cards at the 6 month and 12 month assessments for a total of $50 incentive. They will also keep the provided intervention tools (mobile device, music, and either game app or monitoring app) as an additional incentive. Participants will receive iPod Touch devices to solve problems related to smartphones. iPods function more like a “Walkman” or miniature tablet than a phone, do not receive phone calls that could harass users, and contain enough space to include both very large game apps and music files. They are thin, easily fitting in a purse, bag, or sport armband. All participants will be instructed to either use their personal Wi-Fi Internet access or to travel to a Wi-Fi hotspot once per week to ensure that we receive information from their apps. In the Galveston and Houston areas, free Wi-Fi hotspots are common in coffee shops, fast food restaurants, and in commercial and tourist areas. Participants wishing to use their own phones (Android or iOS) rather than the iPods will be provided with the iPod, but will also be given credit to either the Google Play or iTunes stores and shown how to download the app to their phone. Due to the iPod’s small physical size, large data capacity, and different perceived utility as compared to a phone, we anticipate that the majority of participants will choose to use the iPod. However, this choice should improve acceptability. Participants will be allowed to use their own smart device if they find it more convenient.

Phone counseling will be conducted by three interventionists, all trained by a doctoral-level behavioral scientist using standardized procedures (including role-play and supervised calls with feedback). The counseling prompts are highly standardized, as discussed below, and extensive notes will be taken in phone logs to ensure quality assessment and treatment fidelity. In addition to the topic-based content for each group, counselors will provide feedback on whether the weekly goal was met, ask and record why if it was not met, and inquire about any possible adverse events during the last week.

#### Intervention overview

The two interventions compare the effectiveness of an enhanced intervention to an exemplar of current intervention strategies. Thus, the two interventions share many behavior change strategies by design. The enhanced intervention includes the self-regulatory techniques of the standard intervention and adds techniques associated with play and narrative. In both groups, techniques will be delivered by both the apps and counseling. Table 2 shows the behavior change techniques targeted by the two interventions and their delivery mechanism.

**Table 2:** Behavior change techniques in the two interventions

Behavior change technique	Narrative game intervention	Self-monitoring intervention
Goal setting (behavior)	App + counseling	App + counseling
Problem solving	Counseling	Counseling
Action planning	Counseling	Counseling
Review behavior goal(s)	Counseling	Counseling
Feedback on behavior	App + counseling	App + counseling
Self-monitoring of behavior	App	App
Social support (emotional)	App + counseling	
Information about health consequences	Counseling	
Information about others’ approval	App	
Behavioral practice/rehearsal	App + counseling	
Non-specific reward	App	App
Identification of the self as a role model	App + counseling	
Framing-reframing	App + counseling	
Identity associated with changed behavior	App + counseling	
Verbal persuasion about capability	App	
Mental rehearsal of successful performance	App + counseling	
Focus on past success	App	App
Vicarious consequences	App	

#### Self-monitoring Intervention

**Fig. 3:**
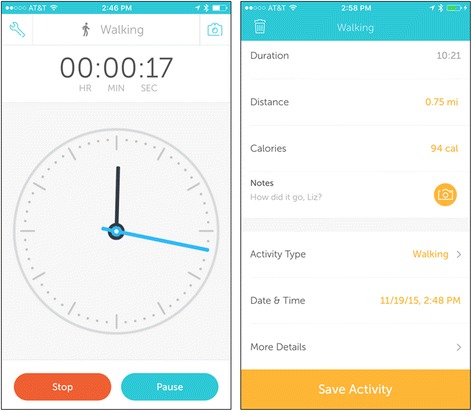
Screen shots from *Runkeeper* app

Participants randomized into the self-monitoring intervention will receive a walking app preloaded onto an iPod touch device, headphones and an armband for using the device, and $50 in credit towards purchasing music and/or additional in-app features. The app *Runkeeper* (FitnessKeeper, Inc., Boston, MA) was chosen because it includes the self-regulatory tools typical of walking/running apps without additional game- or narrative-related components. Users set up music playlists to listen during walks and indicate when they wish to begin and end their walk. On the iPod, the app measures length of time as the primary indicator of activity. The screenshots in Fig. 3 show the walking screen and a walk log from the app. Participants may also enter additional self-reported information, such as their mood upon completing the walk. The app will be configured to send information to individual *Runkeeper* accounts that “friend” the investigator account for the purposes of monitoring participant activity.

Counseling will include standard self-regulatory skill building content from interventions based on Social Cognitive Theory, addressing behavior change techniques related to goal-setting, self-monitoring, feedback, problem solving, and action planning. In order to ensure that equal attention is offered to both intervention groups, counselors will spend additional time with the self-monitoring group participants by discussing monitoring and feedback topics in greater depth and with specifics related to the app (e.g., longer discussion of comparisons to past performance along with a guided tour through the app’s walking logs). The purpose of the extra time spent with these participants is to match the time spent in the narrative and game group discussing narrative-specific topics.

#### Narrative & game intervention

The narrative and game intervention seeks to test the additive impact of narrative and game aspects to a standard self-regulatory intervention. Thus, this intervention is not testing the efficacy of a particular active video game. Rather, it uses the game as well as adapted counseling content to test the feasibility and efficacy of delivering additional behavior change techniques using narrative and game mechanics.

Participants randomized into the narrative game intervention will receive a narrative-based mobile game preloaded onto an iPod Touch device, headphones, an armband for using the device, and $55 in credit towards purchasing music and/or additional in-game features. Table 3 displays how the game and counseling used in the experimental condition will manipulate the variables of interest from the conceptual model. Behavior change techniques taken from Michie and colleagues’ taxonomy are listed as well as pertinent game mechanics [70, 71]

**Table 3:** Major behavior change techniques, related theoretical constructs and game mechanics, and their implementation in the narrative game intervention

Technique	Related constructs	Related game mechanics	Strategies
Goal-setting (behavior)	Competence	Player-defined goals	Set long-term, short-term, and weekly goals
Feedback on behavior	Competence	Feedback	Run logs, game statistics, virtual supplies received
Self-monitoring of behavior	Competence	Points	App monitors intensity, duration, and frequency of activity
Social support (emotional)	Relatedness	Verbal intangible rewards	Game characters provide encouragement
Information about health consequences	Autonomy	N/A	Counselors detail a solid rationale for behavior change
Information about others’ approval	Competence, Relatedness	Verbal intangible rewards	Game characters encourage and congratulate the player character
Behavioral practice/rehearsal	Playful experience	Role play	Game-based activity is by definition practice
Non-specific reward	Playful experience, autonomy	Pick-ups, task non-contingent rewards	“Memorials” and other special virtual items; random gifts from characters
Identification of the self as a role model	Identification, exercise identity, integrated regulation	Representational agency, role play	Participant role-plays as a strong and important person
Framing-reframing	Identification, playful experience, competence	Role play	Walking is framed as play and performance
Identity associated with changed behavior	Exercise identity, integrated regulation	Character identification, role play	Participant identifies with fit character who uses activity to succeed
Verbal persuasion about capability	Competence, relatedness	Verbal intangible rewards	Characters encourage and congratulate player
Mental rehearsal of successful performance	Playful experience, exercise imagery	Role play	Participant imagines saving kittens, children, etc. via physical prowess
Focus on past success	Competence	Rewards of glory	Run logs, special buildings in base, characters mention past exploits
Vicarious consequences	Playful experience, autonomy, competence	Role play	Observe consequences of player and other characters’ activities

.

**Fig. 4:**
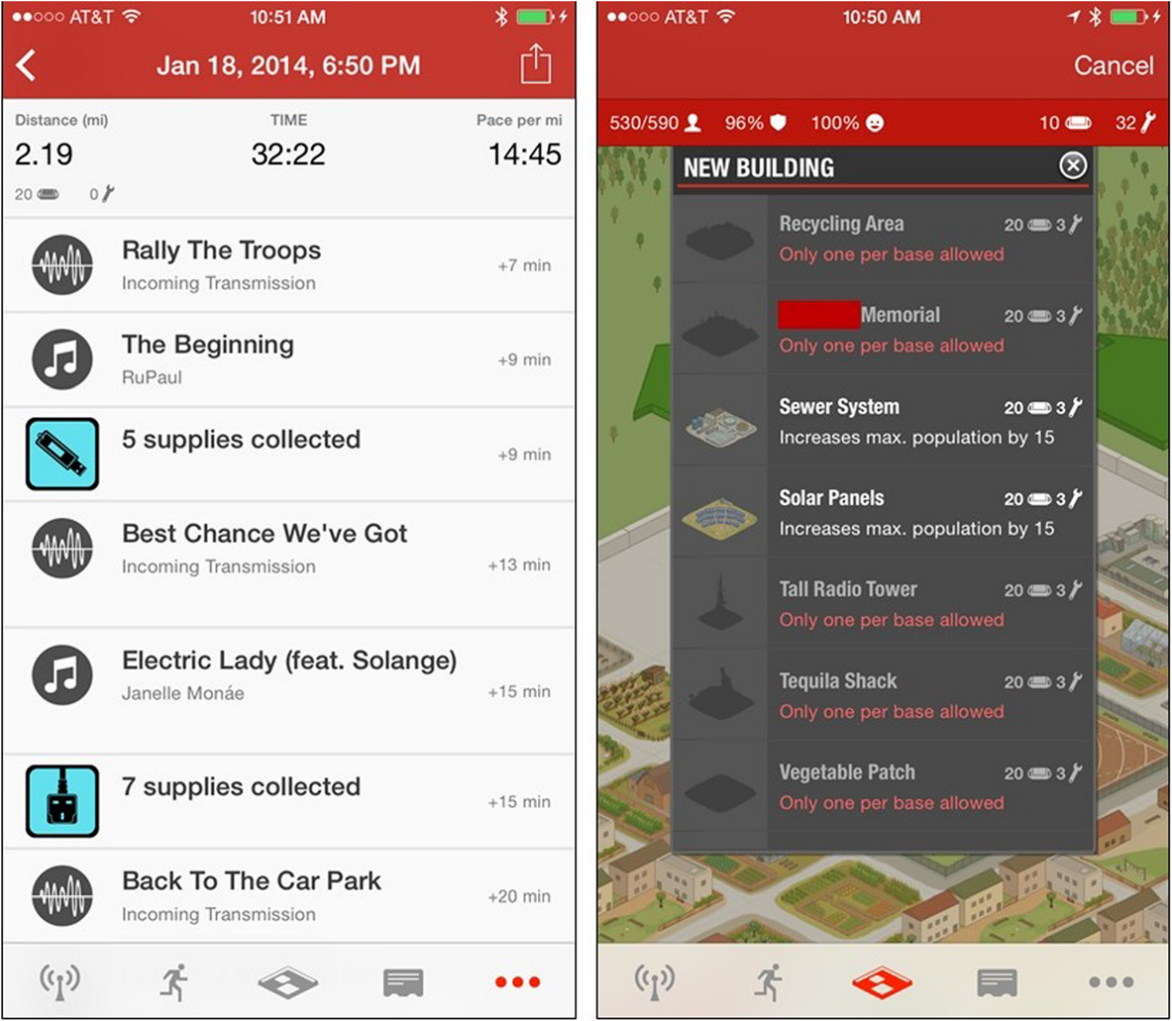
Screen shots from the *Zombies, Run!* game

The game to be used is *Zombies, Run!*, created by developers Six to Start. This game was chosen due to its acceptability in pilot testing, unique blending of an involved narrative storyline with game mechanics, and interactive behavioral tools (e.g., self-monitoring, feedback, goal-setting). *Zombies, Run!* encourages bouts of 30 – 60 minutes of walking, jogging, or running at the player’s preferred pace. Minutes of activity will be the primary measure of activity for this app as well, to prevent possible differences in accelerometer accuracy across devices and apps. Players use the mobile device to set up the game, but during exercise the game is audio-only, with the device held or worn on the arm. The game plays user-chosen music and places clips of an audio narrative in between those songs, to provide an illusion of other characters “radioing in” while the player travels by foot in a post-apocalyptic world. While songs are played, the game provides audio cues when players “pick up” virtual supplies or are chased by zombies. Zombie chases (which can be turned off) prompt players to increase their speed by 20 % for one minute in order to out-run their pursuers. Supplies can be used to build a virtual base, which implements resource management gameplay (planning how to use supplies to create the best base) and provides virtual rewards related to story events (e.g., memorials to fallen characters or buildings specific to new characters’ expertise). The left screenshot in Fig. 4 displays what occurred during one mission. The right screenshot in Fig. 4 shows an example of the buildings available (with the name of the memorial redacted to prevent spoiling the story!).

The player character, referred to as “Runner 5,” is a silent protagonist with no clear age, gender, or race. The game clearly reinforces several of Runner 5’s character traits – heroism, competence, endurance, and loyalty – but leaves the characterization otherwise open to interpretation. As discussed briefly in Table 3, the game uses the words of other characters to demonstrate their esteem for Runner 5 and to provide encouragement.

The game includes over 120 missions in the main storyline across four “seasons,” with side missions that provide additional insight into some of the characters. Additional content is planned for future release, so the storyline will be more than long enough to sustain play without repetition over the year-long period. The narrative is written by a professional writer and includes main characters of multiple genders and races. The self-monitoring and feedback tools provided by the game are very similar to those provided by *Runkeeper*. The core functions (starting a walk, listening to music while walking, ending a walk, viewing feedback afterwards) are also very similar.

Counseling content for the narrative game group was adapted from the narrative counseling used in the PACT study [72] and extended to include appropriate constructs of interest in this study. Table 4 includes examples of counselor prompts. Discussions will draw from the *Zombies, Run!* game and make suggestions related to future play. For example, some sessions will call participants’ attention to the exercise imagery they engage in during walking sessions, and others will encourage participants to imagine their ideal selves when playing as their character. Participants in this group will also receive the basic counseling provided to the self-monitoring group that covers self-monitoring, feedback, action-planning, and problem-solving.

**Table 4:** Examples of counseling content from the narrative group

Concepts	Counseling script suggestions
Games and play	In addition to the game you’ll be playing on your mobile device, let’s think of this whole program as a game. It has a goal, rules, and conflict. How can we make your experience in this program like a well-played game? We’ve set your goals, but what are the rules for reaching them? What conflicts do you think might occur that you’ll need to overcome in order to play well and follow your rules?
Safety	When you decide to play a game, you create what is called a “magic circle.” The magic circle is what makes the game safe. You carve out a little piece of the world and say “here, I can try even the hardest thing, and if I fail it’s not a big deal. I’m trying things out in a safe place.” Do you have any “stretch” goals that are a little harder than your current weekly goals that you’d like to try out, knowing that it’s okay if you don’t reach them?
Identification	When you experience a story, you get to “try on” the characteristics of your character. You play the role of that person. You get to see what it feels like to be that person. What are your impressions of Runner 5? Do you admire her physical strength and endurance? How do you feel when you play as her? What would it feel like if you were more like her?
Exercise imagery	Think about a time when you found exercise really enjoyable and energizing. Imagine what it felt like as vividly as possible. What did you see, and what did you hear? How did you feel physically? How did you feel emotionally? On your next walk, try to keep these feelings in mind as you begin.
Narrative transportation	Let’s try using imagery while you play the game. The next time you play, try to use all of your senses to really feel like you’re experiencing what Runner 5 is experiencing. Create the scene vividly in your mind. What do you see and hear? How does it feel? How do you-as-Runner 5 feel about what is happening?
Relatedness	There are a lot of characters you’ve met in the game. Who do you think is your favorite character? Why? Real life people aren’t the only ones who can give you support and encouragement. How does it feel when Sam and the others praise you? Even though they’re pretend, your feelings when interacting with them are real. Pay attention this week to how the characters make you feel when they talk to and about you.

#### Physical activity goals

In both groups, activity goals will be negotiated between the counselor and the participant. We will recommend a starting goal of 30 minutes of activity at least twice per week, increasing to five times per week by week six. After the first week, participants will increase their goals weekly depending on their preference, increasing goal days, goal minutes, or both.

### Outcome measures

Objective and self-report measures are described below. Assessment times for each outcome are displayed in Table 1.

#### Physical activity

Physical activity will be measured objectively using Actigraph wGT3X BT monitors. Wear time will be seven days at each assessment point. Because continuous measurement is not feasible, a week-long sample will be taken at the three assessment periods. Estimates will be valid if the monitor is worn ≥ 10 hours per day on ≥ 4 days. Non-wear time will be determined by 60 or more of consecutive minutes of zero activity counts. Based on criteria used for the NHANES surveys, epoch length will be one minute, and activity counts over 2020 per minute will be considered moderate-vigorous intensity [73]. Physical activity will be measured as minutes per week of moderate-vigorous intensity activity. A validated and widely used self-report measure (CHAMPS) will also be used to investigate time spent in different activities [74]. The objective measure of minutes per week of moderate to vigorous activity (measured by the Actigraph) at 6 months will be the primary endpoint of the study, with the self-report measure providing additional insight into behavior changes in specific activities and estimated caloric expenditure. CHAMPS is specifically intended for measuring activity among older adults and has been used previously in intervention trials of cancer survivors [75].

#### Secondary outcomes

The Senior Fitness Test will measure fitness (distance walked in six minutes) and function (chair stand, back scratch, chair sit and reach, eight foot up and go) [76]. Height will be measured to the nearest 2 cm without shoes using a standard stadiometer (Seca Corp, Hamburg, Germany). Weight will be taken in light street clothes with empty pockets and with no shoes using a calibrated scale (Tanita, Arlington Heights, IL). The average of two measurements to the nearest 0.1 kg will be used.

Physical (“I have pain”), social (“I feel close to my friends”), functional (“I am able to enjoy life”), emotional (“I feel sad”), and breast-specific (“I am able to feel like a woman”) quality of life will be measured using the 37-item Functional Assessment of Cancer Therapy – Breast (FACT-B) measure [77]. Responses are provided on a Likert scale from 0 (“Not at all”) to 4 (“Very much”). FACT scales have shown excellent reliability and validity across a large number of studies [78]. Subscale Cronbach’s alphas for the FACT-B ranged between 0.63 and 0.86, with a value of 0.90 for the scale as a whole [77].

Other quality of life-related outcomes (depression, anxiety, fatigue, and sleep disturbance) will be measured using PROMIS computer adapted testing [79]. The depression and anxiety scales will be cancer-specific. All item banks for each construct include a number of items and were extensively tested for reliability and validity prior to their implementation. The computer adapted testing procedure uses item response theory to determine which items are shown to participants based upon their previous responses to reduce respondent burden. No more than 12 items per construct will ever need to be answered by participants.

#### Intermediate outcomes

Exercise identity will be measured using the Exercise Identity Scale [80], a 9-item scale with subscales of exercise role identity (“Others see me as someone who exercises regularly”) and exercise beliefs (“Exercise is something I think about often”). Responses range from 1 (“Strongly disagree”) to 7 (“Strongly agree”). This measure is widely used with a reported Cronbach’s alpha of .94, and several factor analyses have confirmed this factor structure [81, 82].

Motivation will be measured using the 19-item Behavioral Regulation in Exercise Questionnaire-2, which measures external (“I exercise because other people say I should”), introjected (“I feel guilty when I don’t exercise”), and identified regulation (“I value the benefits of exercise”), amotivation (“I don’t see why I should have to exercise”), and intrinsic motivation (“I exercise because it’s fun”) [83]. We will also include four items to measure integrated regulation (“I consider exercise a fundamental part of who I am”), which is not traditionally included in exercise measures but which has been found to be an important predictor of exercise behavior [84]. Responses are provided on a 5-point Likert scale from 0 (“Not true for me”) to 4 (“Very true for me”). The complete questionnaire with integrated regulation questions has shown reliability (between 0.70 and 0.93 for the subscales) and predictive validity [84].

#### Other variables

We will measure several other variables in the conceptual model that relate to games and narrative. Narrative transportation in the narrative group will be measured using Green and colleagues’ Narrative Transportation Scale [45]. A 15-item version will be used, which will include four items related to “imagery” that are specific to characters in the game (“While playing the game, I had a vivid image of Sam Yao”) as well as the standard 11 items relating to cognitive (“I was mentally involved with the game while playing it”) and affective (“The game affected me emotionally”) aspects of transportation. Responses will be provided on a seven-point Likert scale with anchors “very much” and “not at all.” This scale is widely used and has demonstrated reliability (alpha > .70) in previous experiments using video games [85] and diverse samples of adult women viewing cancer-related narratives [53]. Only participants in the narrative game group will complete this scale, as its questions do not make sense in the absence of a storyline. We will pilot test use of the Presence Self-Assessment Manikin as a crude indicator of immersion applicable to both applications [86]. This measure uses pictures to represent feeling “part of the action” in a specific medium (human figure inside a TV) to removed from the action (human figure far away from a TV).

The Exercise Imagery Questionnaire is a widely-used measure that includes three subscales: appearance (“I imagine a leaner-me from exercising”), techniques (“When I think about exercising, I imagine my form and body position”), and energy (“To get me energized, I imagine exercising”) [87]. An additional subscale related to enjoyment (“When I think about exercise, I imagine myself having fun while exercising”) will be included [47]. The resulting scale has 12 items and responses range from 1 (“Never”) to 9 (“Always”). Cronbach’s alphas ranged between 0.79 and 0.93 for the four subscales [47].

Character identification cannot be accurately compared across groups, as the monitoring app does not include characters. To provide a proxy for character identification, we will use the procedure described by Przybylski and colleagues for investigating discrepancy between the ideal self and self while playing the game/using the app [49]. Participants will complete the Ten Item Personality Inventory with reference to their ideal self (at baseline), then regarding themselves while using the app (other time points). Correlations between ideal self and game-self for each of the items will be averaged, with neuroticism items reverse-coded, to create a number between −1 and 1 that indicates ideal-self and walking-self convergence. This convergence will be considered a proxy measure of the extent to which identification with the player character led to “trying on” of her characteristics.

The Play Experience Scale is a 16-item scale made up of four subscales: freedom (“I was able to make the game do what I wanted it to”), no extrinsic (“I was not worried about someone judging how I performed in the game”), play-direct (“I would characterize my experience with the game as playing”), and autotelic-focus (“When I was using the game, I was focused on the task at hand”). Responses range from 1 (“Strongly disagree”) to 6 (“Strongly agree”). This is a relatively new scale with few published studies using it, but it has demonstrated reliability (alpha > .80) and convergent validity with intrinsic motivation (r = .79) [55].

The Basic Psychological Needs in Exercise Scale will measure autonomy (“I feel that the way I exercise is the way I want to”), competence (“I feel that exercise is an activity which I do very well”), and relatedness (“My relationships with the people I exercise with are very friendly”). Responses to this 11-item scale use a 1 – 5 Likert scale (“I don’t agree at all” to “I completely agree”). Reported alphas for the subscales were 0.75 or higher [88]. This scale has been used similarly in past studies, for example to investigate the relationship between perceived competence and exercise role identity (partial r = 0.20) [81].

At baseline, demographic information will be collected regarding gender, race, and age. Cancer-specific information will also be collected regarding time since diagnosis, type/stage of breast cancer, and type of treatment.

#### Acceptability and process measures

Acceptability items will be adapted from Vandelanotte and colleagues [63, 64]. Items will measure aspects of acceptability for both the device (e.g., “I found the device very cumbersome to use,” and “I think I would need the support of a technical person to be able to use this device”) and the applications (e.g., “I think the app is user-friendly” and “it was convenient for me to use the application”). Process measures for both groups will include logs of completed counseling phone calls, time spent in each call, attrition, and adverse events related to participation. Brief specific questionnaires (including measures of narrative transportation and identification discussed above) will be provided for participants in the narrative group to complete immediately after the first mission and specific highly emotional missions (“A Voice in the Dark” and “Alternates”), to determine the acceptability of content. Objective process measures will also be recorded from the two apps. *Zombies, Run!* will send exercise session information to the *Runkeeper* service, where an interventionist account will “friend” the participant’s account. Number of walks per week and total minutes per week will be abstracted from the website. Thus, we will be able to investigate both overall lifestyle activity (Actigraph) as well as activity specifically related to the apps.

During the 12 month visit, we will conduct brief interviews with all participants regarding their opinions on the apps and counseling sessions. These discussions will be audio recorded, transcribed, systematically coded using thematic analysis procedures [62], and used to better understand the acceptability of different intervention components.

Adverse events will be tracked throughout the study via direct inquiry during each counseling call, direct inquiry during assessment visits, and review of information taken from the apps that may indicate dangerous activities (e.g., over-exertion). Call scripts/log sheets will include prompts for counselors to inquire about a list of anticipated adverse events (muscle pain, chest pain, etc.) as well as to ask open-ended questions about any other possible adverse events in the past week. Information on adverse events, whether they are related to the study, their descriptions, any participants who withdraw due to the events, and risk of events in each group will be collected. We will use the National Cancer Institute common terminology criteria for adverse events grading scale (from a score of 1 for mild events to 5 for death related to the event) [89]. The study physician (Dr. Volpi), who will be blinded as to group assignment, will make attributions as to whether events are related to the study.

### Data management and analysis

#### Sample size and power

This study is powered to detect differences between the two groups in minutes of moderate-vigorous physical activity at 6 months. We used SAS 9.4 (SAS, Inc., Cary, NC) to calculate sample size based on several studies similar to this one. Similar trials of telephone counseling and activity monitoring among breast cancer survivors produced effect sizes ranging from 0.53 to 0.82 [90–92]. This study includes an alternative intervention rather than a true control group, so it is likely that the alternative group will show improvements as well. We anticipate that improvements in the other group will be weak at 6 months due to the absence of motivation-related intervention content. We thus have powered for a conservative effect size for physical activity at 6 months of approximately d = 0.65, which would require an N of 78 for 80 % power at an alpha of 0.05. This effect size translates to a between groups difference of approximately 20 minutes per week (SD = 30 minutes). Assuming a correlation between baseline and follow-up physical activity of approximately r = .65 (based on the pre-experimental pilot study), the required N would be even smaller, approximately 50.

Similar studies that investigated maintenance are few and show disparate results; thus, power was difficult to calculate. For example, Rogers et al. reported large differences in change scores between groups from baseline to the end of maintenance (79 vs. -22 minutes per week; 3 month follow-up) [93], whereas Vallance et al. reported smaller differences (24 vs. -14 minutes per week; 6 month follow-up) [94]. Assuming large standard deviations for these change scores, we plan to recruit a sample of 120. This sample should be sufficient to detect group differences during the intervention and maintenance period even in the case of heavy attrition.

#### Analytic plan

This study is an evidentiary study [95], with behavior as the outcome of interest. Thus, the primary endpoint of this study will be behavioral change in minutes of objectively-measured moderate-vigorous physical activity at 6 months. We will use an analysis of covariance to test the difference between groups at 6 months, controlling for baseline. A secondary analysis will use linear mixed modeling to investigate changes in minutes of objectively-measured moderate-vigorous intensity physical activity over time at baseline, 6, and 12 months. All analyses will follow the intent to treat principle and will be conducted after assessing measure reliability, variable distributions, and outlying observations. Missing data will be imputed using multiple imputation, and significance will be set at an alpha of 0.05. Effect sizes for the difference between the two groups will be calculated for all models run. Analyses will be performed using SAS 9.4 (SAS, Inc., Cary, NC). Age, race, and time since cancer diagnosis will be included as covariates, and both adjusted and unadjusted results will be investigated [96]. We will also use Student’s t and chi square tests to compare those who complete the study to drop-outs.

We will follow the same analysis procedures for the health outcomes (fitness, function, weight, quality of life, depression, fatigue, anxiety, and sleep). For the intermediate outcomes, we will conduct exploratory investigations using correlation analyses and the ANCOVA procedures discussed above. Because so little is known about potential effect sizes for variables such as character identification and exercise imagery in these contexts, simple exploratory tests appear most appropriate to inform mediation analyses in future studies. Results of these investigations will be used for refining the conceptual model for eventual testing in a larger trial.

For the qualitative interviews, we will combine grounded theory with an overall theory-based framework to conduct the thematic analyses [62]. That is, theory will guide the questions asked and the initial codes used to analyze the transcripts. While coding the transcripts, grounded theory will guide development of additional codes based upon themes that arise from participants’ words. We have previously used this strategy to investigate specific questions about theoretical constructs as well as discover unexpected reactions [97].

## Discussion

To improve the health of breast cancer survivors, novel intervention strategies that can increase physical activity and sustain those increases over time are required. This trial will compare the effectiveness of two novel, technology-based interventions, each with the capacity for large-scale improvement of breast cancer survivors’ health. This comparison builds upon several years’ worth of formative data, including focus groups and pre-experimental pilot tests.

By comparing the two interventions, we will test the additive benefit of a focus on narrative and games over and above the benefit of a basic self-monitoring intervention. We hypothesize that a combination of a narrative-based active video game and counseling that reinforces persuasive elements of the game will increase exercise identity, intrinsic motivation, and autonomous forms of extrinsic motivation such as integrated and identified regulation. Identity and autonomous motivation are powerful theoretical constructs that we hope can produce sustained, long-term change in exercise behavior patterns.

This protocol has several limitations. By using commercial technology, we cannot control changes to the apps over time. Large-scale changes to either app are unlikely, as their core components have not changed over the past three years, and similar apps exist that could be used if necessary. Some participants may not enjoy the zombie theme of the narrative game; in this case, we have made a list of missions that tell stories with little violent or horror content.

Even in the case of null results, this trial will produce a large amount of illuminating data. Investigators will be able to closely monitor exercise behavior in both groups for as long as participants use the apps. If and when participants stop using the apps is very useful information regarding the life cycle of app use. Little is known about how active game or activity monitor use changes over extended periods of time. Measurement of many potential mediating variables and process measures will help answer not just whether but how and why the interventions are successful.

## Additional files

Additional file 1: CONSORT 2010 checklist of information to include when reporting a randomised trial. (DOC 218 kb)

## Abbreviations

Cm: centimeter
FACT-B: Functional Assessment of Cancer Therapy – Breast
Kg: kilogram
NHANES: National Health and Nutrition Examination Survey
NTT: narrative transportation theory
SDT: self-determination theory
